# Optical limitations guide behavioral algorithms in *Drosophila*

**DOI:** 10.3389/fnbeh.2014.00374

**Published:** 2014-10-31

**Authors:** Jessica L. Fox

**Affiliations:** Department of Biology, Case Western Reserve UniversityCleveland, OH, USA

**Keywords:** *Drosophila*, saccades, optic flow, visual acuity, halteres, walking, head body coordination

Vision is complicated by movement. As a seeing animal moves through space, its own movement causes the entire visual scene to move on the retina, creating a wide-field motion stimulus that may be irrelevant and confounds the visual system's ability to form a stable image. In both vertebrates and invertebrates, self-motion of the body is detected by mechanosensors (in vertebrates, the inner ear; in invertebrates, statoliths, or other organs), and countered by compensatory rotations of the eye that steady the image on the retina. These counter-rotations remove the irrelevant stimulus and allow the animal to focus on motion in the external environment. But what happens when the animal intentionally shifts its gaze to another part of its visual world?

In voluntary eye movements to fixate objects of interest, the eye is moved as fast as possible to minimize the time during which the visual image is blurred. These rapid movements are known as saccades, and they occur in human eyes so quickly that we don't notice them as we move our eyes across a page of text or a visual scene. Insects perform the same kind of fast fixating saccades, but since their eyes are fixed in their heads, their saccades take the form of full body rotations, which change the direction of movement. These body saccades serve multiple purposes. They minimize the time of motion blur as they do in vertebrates, and by minimizing the time spent in rotation they allow the fly to rapidly change heading. Because many insects lack stereoscopic vision for depth perception (Geurten et al., 2014), self-motion is also essential to obtaining depth information. Since translatory motions provide more distance cues than rotational motions, separating body movements into long periods of pure translation, interspersed with fast periods of pure rotation, is an effective method for organizing visually-guided self-motion. Previous studies have found that fruit flies use this strategy when flying (Wolf and Heisenberg, 1980). However, when walking, their forward speed is lower, and the demands on visual control systems are quite different from those in flight. Do flies also follow this motion-vision strategy while they walk?

A recent paper by Geurten et al. (2014) indicates that the algorithm for organizing visually-guided flight is also followed on the ground. Using videos of walking flies and k-means clustering, Geurten et al. sorted fly walking behaviors into several prototypical movements (PMs). They find that walking flies, like flying flies, have a small number of distinct PMs, with different amounts of time spent in each. The flies show longer periods of translatory (straight) movement, interspersed with short times spent in rotational saccades (as well as rest periods—which is how the flies spend the majority of their time). Walking saccades are slower than those during flight, but are similar in duration and amplitude to those observed in walking blowflies (*Calliphora*), and bees (*Apis*). Thus, at first glance, *Drosophila* walking behavior appears to follow the same saccade algorithms seen in other walking insects (Blaj and van Hateren, 2004).

However, there are two means by which an insect can make a saccade, with very distinct sensorimotor consequences for each: the insect can rotate its entire body and change its heading, or it can use its highly flexible neck to simply rotate its head. In bees and blowflies, both kinds of saccades are observed, but Geurten et al. observed that the rotational saccades of fruit flies are not accompanied by independent head saccades. Why not?

The answer may lie in the optics of the eyes. The spatial and temporal resolution of the eyes of *Drosophila* is significantly lower than that of the eyes of *Apis* or *Calliphora* (Laughlin and Horridge, 1971; Petrowitz et al., 2000). Geurten et al. modeled the effects of a head saccade on the visual scene by processing several different images with a filter based on the measured optics of the fly's eye, to gain an understanding of what the fly sees. They found that *Drosophila* would have to move its head well beyond the physically possible range to obtain an image shift comparable to those observed from *Calliphora* or *Apis*. Thus, *Drosophila* turns its entire body rather than its head.

How does this compare with fruit fly head movement behavior during flight? A satisfactory answer is not yet available. The heads of freely-flying fruit flies are difficult to resolve in video, so we have resorted to tethered flight as a substitute. In flying flies that cannot rotate their bodies, head saccades are prevalent, and the head closely follows moving wide-field visual stimuli to stabilize it on the retina (Fox and Frye, 2014). In other experiments, larger flies (*Calliphora* and *Musca*) were tethered to a pin such that they could freely rotate within a circular visual display (Land, 1973; Geiger and Poggio, 1977; Bender and Dickinson, 2006), or magnetic coils were used to observe head movements in free flight (Schilstra and van Hateren, 1998; van Hateren and Schilstra, 1999). Although there is some disagreement in those studies about whether flies move their heads independently of their bodies during flight, or whether the observations in larger flies are applicable to fruit flies, it is clear that all flies perform saccades with the entire body. These saccades are visually initiated, but their duration and magnitude is determined by feedback from the mechanosensory halteres (Bender and Dickinson, 2006), which are oscillated in flight and detect body rotations.

Geurten et al. note that *Drosophila's* lack of head rotations in walking is accompanied by a lack of haltere movements during walking. This is in sharp contrast to the walking behavior of *Calliphora*, which performs both head saccades (Blaj and van Hateren, 2004), and haltere oscillations (Sandeman and Markl, 1980) during walking behavior. Although this data is only correlative for now, it is an important observation that underscores the diversity of fly behaviors. This study suggests that there may be multiple strategies for gaze control during walking in dipteran insects: one in which a high-resolution visual system is rotated rapidly under the influence of the oscillating halteres, as in *Calliphora*, and another in which a lower-resolution system remains more or less fixed to the rotating body without haltere input. Their study raises important questions about both the distinction between these strategies, and how the overall circuitry of the fly's sensorimotor system might be adapted to particular sensory, contextual, and ecological constraints.

## Conflict of interest statement

The author declares that the research was conducted in the absence of any commercial or financial relationships that could be construed as a potential conflict of interest.
